# Recombinant human epidermal growth factor combined with vacuum sealing drainage for wound healing in Bama pigs

**DOI:** 10.1186/s40779-021-00308-5

**Published:** 2021-03-09

**Authors:** Shuai Wei, Wei Wang, Li Li, Hao-Ye Meng, Chun-Zhen Feng, Yu-Ying Dong, Xi-Chi Fang, Qi-Qiang Dong, Wen Jiang, Hai-Li Xin, Zhan-Zhen Li, Xin Wang

**Affiliations:** 1 Institute of Orthopaedics, Beijing Key Lab of Regenerative Medicine in Orthopaedics, Chinese PLA General Hospital, Beijing, 100583 China; 2 Zhoushan Dinghai Guanghua Hospital, Zhoushan, 316000 China; 3 Tianjin Hospital, Tianjin University, Tianjin, 300211 China; 4 Geriatric Neurological Department of the Second Medical Center & National Clinical Research Center for Geriatric Diseases, Chinese PLA General Hospital, Beijing, 100853 China; 5 Department of Orthopedics, Traditional Chinese Medical Hospital of Xinjiang Uygur Autonomous Region, Urumqi, 830000 China; 6 Department of Stomatology, Chinese PLA General Hospital, Beijing, 100853 China; 7 Department of Plastic Surgery, General Hospital of Taiyuan Iron and Steel Limited Company, Taiyuan, 030009 China; 8 Hand Microsurgery Department, Shenzhen People’s Hospital, Shenzhen, 518020 China; 9 Third Surgery Department, Zhengzhou Renji Hospital, Zhengzhou, 450000 China; 10 Department of Orthopedics, the First Affiliated Hospital of Medical College, Shihezi University, Shihezi, Xinjiang, 832000 Uygur Autonomous Region China; 11 Pharmacy Department, Chinese PLA General Hospital, Beijing, 100853 China

**Keywords:** Vacuum sealing drainage, Epidermal growth factor, Skin wound healing, Full-thickness skin defect

## Abstract

**Background:**

Vacuum sealing drainage (VSD) and epidermal growth factor (EGF) both play an important role in the treatment of wounds. This study aims to explore the effects of the combination of VSD and EGF on wound healing and the optimal concentration and time of EGF.

**Methods:**

We tested the proliferation and migration capacity of HaCaT and L929 cells at different EGF concentrations (0, 1, 5, 10, and 100 ng/ml) and different EGF action times (2, 10, and 30 min). A full-thickness skin defect model was established using male, 30-week-old Bama pigs. The experiment included groups as follows: routine dressing change after covering with sterile auxiliary material (Control), continuous negative pressure drainage of the wound (VSD), continuous negative pressure drainage of the wound and injection of EGF 10 min followed by removal by continuous lavage (V + E 10 min), and continuous negative pressure drainage of the wound and injection of EGF 30 min followed by removal by continuous lavage (V + E 30 min). The wound healing rate, histological repair effect and collagen deposition were compared among the four groups.

**Results:**

An EGF concentration of 10 ng/ml and an action time of 10 min had optimal effects on the proliferation and migration capacities of HaCaT and L929 cells. The drug dispersion effect was better than drug infusion after bolus injection effect, and the contact surface was wider. Compared with other groups, the V + E 10 min group promoted wound healing to the greatest extent and obtained the best histological score.

**Conclusions:**

A recombinant human epidermal growth factor (rhEGF) concentration of 10 ng/ml can promote the proliferation and migration of epithelial cells and fibroblasts to the greatest extent in vitro. VSD combined with rhEGF kept in place for 10 min and then washed, can promote wound healing better than the other treatments in vivo.

## Background

Because of the long treatment cycle of various accidents and the large investment of medical resources, the burden of treating skin defects is increasing rapidly [1, 2]. Therefore, for patients, medical workers and society, it is urgent to develop a simple and effective treatment that can shorten the healing time. The wound healing process is a complex biological event that includes inflammation and the proliferation and migration of different cell types, and its aim is to restore the physiological and barrier functions of the skin, and then control infection [3]. At the same time, there are several important and orderly physiological events in the healing process, such as extracellular matrix (ECM) synthesis, neovascularization [4], and collagen secretion and deposition leading to re-epithelialization and the formation of granulation tissue [5].

For the wound healing process, recent studies have shown that some new technologies had successfully accelerated wound healing [6, 7]. In the treatment of diabetic wounds, Dwivedi et al [6] successfully designed a dual-carrier nano scaffold that can accelerate wound healing, which can simultaneously release recombinant human epidermal growth factor (rhEGF) and gentamicin sulfate. At the same time, some innovative traditional treatment techniques are also playing an important role, especially vacuum sealing drainage (VSD). VSD is a kind of medical technology that uses vacuum dressing to accelerate wound healing, and it has been approved and used clinically for many years [8–10]. Compared with traditional treatment methods for wound healing, the clinical effects of VSD are obvious and promising [11, 12]. VSD is applied as a special foam dressing and a drainage tube to fill or cover the wound of a patient with skin or tissue defects. Then, the wound surface and dressing are sealed with a bio-semipermeable membrane to form a closed micro-environment, and the drainage tube and vacuum source are connected to establish a controlled negative pressure [13]. The harmful gases produced by the decomposition of necrotic tissue around the wound can penetrate the outer layer of the semipermeable membrane, but the bacteria outside the membrane cannot enter the wound surface [14]. At the same time, the decomposed necrotic tissue can be discharged through a negative pressure drainage tube. In general, VSD can significantly improve the blood circulation of wounds, thereby reducing tissue oedema and bacterial colonization [15], promoting the growth of granulation tissue and improving the wound healing rate. The negative pressure of the VSD can greatly inhibit the growth of bacteria and reduce the need for antibiotics [16], by producing a relatively hypoxic environment on the wound surface without affecting the surrounding healthy tissues. However, we have often found that more necrotic tissue from deep wounds will soon be distributed in the VSD, especially when the wound is seriously polluted; at this time, it is necessary to replace the negative pressure suction device or rinse it. Epidermal growth factor (EGF) solution shows promising clinical prospects as the flushing solution for negative pressure suction devices.

EGF is a kind of multifunctional cell growth factor produced by platelets, macrophages and monocytes [17], that plays an important role by binding to epidermal growth factor receptor (EGFR) on cell surface [18]. EGFR is expressed on various cell surfaces, including fibroblasts, endothelial cells and smooth muscle cells, and especially, epidermal cells [19]. After binding with EGF, the intrinsic protein tyrosine kinase activity of EGFR is stimulated, thus stimulating a cascade of signal transduction, leading to a variety of biochemical processes in cells. Receptor tyrosine kinases (RTKs) can control various cellular functions through multiple signalling mechanisms; RTK signal attenuation is mainly achieved by endocytosis, which can remove RTK from the cell surface [20]. Among the RTK signals in cells, EGFR is the most characteristic. As a result, the activated signal of EGFR will eventually be transmitted to the nucleus, thus promoting DNA synthesis and cell proliferation and regulating cell metabolism [21, 22]. In particular, the activated signal of EGFR can also promote chemotaxis and the reconstitution of cells, thereby promoting granulation tissue and epidermis formation [23]. However, the exudate of the wound only contains a low level of growth factor, and a certain level of cathepsin, which can destroy the structure of the growth factor, leading to poor wound healing [24]. Therefore, it is advisable to use EGF as a flushing solution when applying VSD to a wound healing treatment. At present, rhEGF has been used in clinical medicine for more than 20 years [25]. A new study has shown that EGFR has different fates through different internalization pathways under different concentrations of EGF and is internalized and transported to the surface of the cell to maintain sustained signalling or enter the lysosome, where it is degraded and inactivated [26]. There is no relevant study on the optimal concentration and time of rhEGF for use as a flushing fluid for VSD.

Pig skin contains a lower elastic fibre content than human skin or an underdeveloped sub-cutaneous plexus [27]. However, compared with most small mammals, in terms of healing by the contraction exerted by the panniculus carnosus, pig skin has many similarities with human skin, such as wound healing primarily via reepithelization, a thick epidermis, rich subcutaneous adipose tissue and a similar collagen composition [28]. In the present study, we evaluated the efficacy of rhEGF combined with VSD for wound healing in Bama pig; and provide some constructive suggestions for the optimal clinical application of rhEGF as a flushing fluid for VSD.

## Methods

### Acquisition of fibroblasts and epidermal cells

The fibroblasts used in this experiment are from the L929 cell line (Cobioer No.: CBP60878), which was purchased from Cobioer Biosciences Co., Ltd., Nanjing, China (http://www.cobioer.com). The cell line was preserved in liquid nitrogen. After cell resuscitation, the cells were inoculated in high-sugar DMEM supplemented with 10% fetal bovine serum (FBS) and, were then cultured in an incubator at 37 °C under an atmosphere of 5% CO_2_. The culture medium was replaced every 2 days, and the cell status was observed and recorded. When the cells adhered to the bottom of the whole culture bottle, they were passaged. When the cells were spindle-shaped and grew well without cell debris, they were cultured for reserve.

The epidermal cells used in this experiment were from the HaCaT cell line (Cobioer No.: CBP60331), which was also purchased from Cobioer Biosciences Co., Ltd., Nanjing, China (http://www.cobioer.com). After cell resuscitation, the cells were inoculated in low-sugar DMEM supplemented with 15% FBS and; were then cultured in an incubator at 37 °C under an atmosphere of 5% CO_2_. The culture medium was replaced every 3 days, and the cell status was observed and recorded. When the cells adhered to the bottom of the culture bottle, they were passaged. When the cells were island-like shaped and grew well without cell debris, they were cultured for reserve.

### Influences of rhEGF on the proliferation of fibroblasts and epidermal cells by the CCK-8 assay

The reserved L929 cells were resuspended in high-sugar DMEM supplemented with 10% FBS and 1% penicillin/streptomycin to a concentration of 1 × 10^4^ cells/ml. The cell suspension was immediately inoculated into 96-well plates (200 μl per well) and then incubated in an incubator for 12 h. After the cells were attached, they were divided into five groups: Normal, 1 ng/ml rhEGF, 5 ng/ml rhEGF, 10 ng/ml rhEGF and 100 ng/ml rhEGF. Cell-free medium (200 μl per well) was used as a blank control. rhEGF was diluted to a specific concentration using cell culture medium, which was replaced every 2 days. rhEGF was provided by Huashengyuan Genetic Engineering Development Co., Ltd., Shenzhen, China (http://szshsyjygcfz.yixie8.com/). CCK-8 reagent (CA1210, Solarbio, Beijing, China) was used to detect the proliferation of L929 cells on the 1st, 3rd, 5th and 7th days of culture. Briefly, the medium was replaced with 200 μl per well of fresh medium with 20 μl CCK-8 reagent. Then, each group of L929 cells was placed in a cell incubator for 2 h and evaluated with a trace orifice spectrophotometer (Epoch Take 3, BioTek) to measure the absorbance at 450 nm. All measurements were repeated three times independently and the blank control value was subtracted from each set of experimental data.

According to the above experimental procedure for L929 cell proliferation, we also determined the proliferative activity of L929 cells under different rhEGF stimulation times of 2, 10 and 30 min at a set concentration of 10 ng/ml. Briefly, after cell adherence to 96-well plates, 3 groups of cells were stimulated with 10 ng/ml rhEGF for a specific time, and the medium was then replaced with fresh medium without rhEGF. The following operating procedures were the same as above, and cell proliferative activity was measured on the 1st, 3rd, 5th and 7th days of culture. Meanwhile, in the same way, the proliferative activity of HaCaT cells was measured at different rhEGF concentrations and different rhEGF stimulation times.

### Influences of rhEGF on the migration of fibroblasts and epidermal cells by the scratch test

The reserved L929 cells were resuspended in high-sugar DMEM supplemented with 10% FBS and 1% penicillin/streptomycin to a concentration of 5 × 10^5^ cells/ml. The cell suspension was immediately inoculated into 6-well plates (2 ml per well) and then incubated in an incubator. After cells were attached and reached 95% confluency, cells in every well were scraped in a straight line to create a scratch with a P200 pipette tip. Then, the exfoliated cells were washed three times with sterile PBS, and the wells were filled with different concentrations of rhEGF solution at 0, 1, 5, 10 and 100 ng/ml. Next, each group of L929 cells was placed in a cell incubator for 24 h and was then removed to take-photographs using an inverted microscope.

According to the above experimental procedure for L929 cell migration, we also determined the migration activity of L929 cells under different rhEGF stimulation times of 2, 10 and 30 min at a set concentration of 10 ng/ml. Briefly, after cell adherence to 6-well plates, 3 groups of cells were stimulated with 10 ng/ml rhEGF for a specific time, and the medium was then replaced with fresh medium without rhEGF. The following operating procedures were the same as above, and the cell migration activity was measured at 24 h of culture by taking photographs using an inverted microscope. Meanwhile, in the same way, the migration activity of HaCaT cell was measured at different rhEGF concentrations and at different rhEGF stimulation times.

### Animal treatment

This study was approved by the Ethics Committee of the Laboratory Animal Research Center of the First Clinical Center, Chinese PLA General Hospital (Approval No. 2016-× 9–07), and animals were handled according to international animal welfare standards. Nine male, 30-week-old Bama pigs, weighing 19.0–28.5 kg, were purchased from the Animal Center of Taizhou Taihe Biotechnology Co., Ltd. (license No. SYXK (Su) 2018–0035); and raised in a sterile environment in single stainless-steel cages with a length of 1.2 m and a width of 0.5 m. Artificial feeding was implemented, twice a day at 8:30 a.m. and 4:00 p.m.

On the morning of the operation, diet and drinking water were limited, animals were weighed, and their body temperature was measured. Before anesthesia induction, scopolamine (0.01 mg/kg, Suicheng Pharmaceutical Co., Ltd., Henan province, China) was used to inhibit cholinergic activity. The Bama pigs were anesthetized by injection of Zoletil®50 (505 mg/kg, Virbac Group, France) and Lumianning (2 mg/kg, Jilin Huamu Animal Health Products Co., Ltd., Jilin province, China) into the muscles of the buttocks. A portable multi-parameter monitor was used to detect important parameters, such as the blood oxygen saturation, heart rate and respiration of experimental animals. At a later stage, the anesthetics were supplemented according to 1/4 of the amount of the induced anesthetics. Skin was prepared at the surgical sites on both sides of the Bama pig’s spine.

The animals were fixed on the operating table for anesthesia maintenance, and the back of the experimental animal was cleaned. Then, a 15 cm × 10 cm sponge was placed on the back of the experimental animal, and the connecting tube was inserted into the sponge. Auxiliary material was then used to adhere the sealing sponge and to connect the external head of the connecting pipe to the negative pressure device. Methylene Blue Solution (15 ml, G1303, Solarbio, China) was diluted to 500 ml with normal saline. An infusion set and 15 ml syringe were used to connect the tube on the sponge to the dye with 250 ml of diluted Methylene Blue Solution, and then open the VSD negative pressure device and adjust the negative pressure value to − 125 mmHg for suction. At the end of dyeing, the negative pressure device was closed, the auxiliary material was removed, and photos were taken to evaluate the diffusion effect of the Methylene Blue injection and infusion.

The Bama pigs were fixed on the operating table in the prone position, and the operation area of the pig’s back was marked with a marker pen, 15 cm in length × 5 cm in width; one surgical area was on the left, and two surgical areas were on the right (named − 1, − 2, − 3). After sterilization and alcohol deiodination, an operation knife and an electric knife were used to make three wounds on the back of the pig, which were 15 cm in length × 5 cm in width × 1.5 cm in depth in the muscular membrane, and hemostasis was achieved by electrocoagulation. The shortest distance between each wound was 5 cm in each direction to avoid cross contamination. According to the different methods of wound repair, the experimental animals were randomly divided into four groups: Routine dressing change after covering with sterile auxiliary material (Control, 1–1, 1–2, 2–1, 2–2, 3–1, 3–2, the front number is the label of the Bama pig after random coding adjustment), continuous negative pressure drainage of the wound (VSD, 1–3, 4, 5, 6, 7, 8, 9–1), continuous negative pressure drainage of the wound and injection of EGF 10 min followed by removal by continuous lavage (V + E 10 min, 2–3, 4, 5, 6, 7, 8, 9–2) and continuous negative pressure drainage of the wound and injection of EGF for 30 min followed by removal by continuous lavage (V + E 30 min, 3–3, 4, 5, 6, 7, 8, 9–3). After the operation, anti-infective drugs were injected into the muscles, and the Bama pig was transferred to a separate cage after it awakened. In the Control group, the sterile auxiliary material was replaced every 2 days; in the VSD group, the negative pressure drainage tube was washed with normal saline every 2 days to avoid blocking; in the V + E 10 min and V + E 30 min groups, EGF was injected at a volume of 30 ml (4 μg/ml) twice a day, the negative pressure suction was closed, and the VSD was opened 10 min and 30 min later, respectively. At the same time, regularly observe the shape of the sponge at the wound surface and whether there is fluid accumulation under the Auxiliary material, so as to confirm the negative pressure effect of VSD and adjust the negative pressure value.

### Analysis of wound closure and wound healing

According to the experimental procedure, the Bama pigs were anesthetized and euthanized by an intravenous injection of potassium chloride on the 10th day after the operation. The anesthesia method was as described above, and 10 d after the operation, with sterile excipients and the VSD removed, photos were taken with a digital camera directly above the wound. The contractibility rate of the wound = (1 - current wound area/ initial wound area) × 100%; the filling rate of granulation tissue = (1 - current wound volume/ initial wound volume) × 100%; the hydroxyproline content in the wound was calculated according to the Kit instructions (BC0255, Solarbio, Beijing, China).

### Histological assessment of wound healing

The wound tissues were fixed in 4% paraformaldehyde for 2 d at room temperature and were then transferred to PBS buffer and washed 3 times. Next, the wound tissues were placed into an ASP200S automatic tissue dehydrator for automatic dehydration. A BMJ-1 biological tissue embedding machine was used for paraffin embedding, and a Leitz 1516 paraffin tissue slicer was used for 3 μm paraffin sectioning. The sections were baked at 65 °C, dewaxed and rehydrated, and then stained. Hematoxylin and Eosin (HE) staining was performed with a commercial H&E Staining Kit (G1120, Solarbio, Beijing, China): The sections were placed in hematoxylin staining solution for 10 min, rinsed with water, moved to a differentiation solution for 1 min, washed with distilled water for 15 min, moved to an eosin staining solution for 1 min, washed with distilled water for 5 min, subjected to conventional dehydration, and sealed using transparent and neutral resin. Transverse 3-μm-thick paraffin sections of the wound tissues were cut and stained using a modified Masson’s Trichrome Stain Kit (G1345, Solarbio, Beijing, China) and were then subjected to conventional dehydration and transparent and neutral resin sealing. The images were captured by a microscope equipped with a DP71 camera (BX51, Olympus, Tokyo, Japan).

### Immunohistochemical assessment of collagen deposition in wound healing

After dewaxing and rehydration, the sections were treated with 3% hydrogen peroxide for 10 min to quench the endogenous peroxidase. An immunohistochemical pen was used to circle the tissue on glass, and non-specific binding was blocked with 10% goat serum albumin (SL038, Solarbio) for 30 min, after which the samples were washed three times for 5 min each time with PBS. A rabbit anti-Collagen I antibody (1:100, ab34710, Abcam, Cambridge shire, England) and a mouse anti-Collagen III antibody (1:200, ab23445, Abcam, Cambridge shire, England) were applied as the primary antibodies and were incubated in a humidified chamber overnight at 4 °C. The excess primary antibody was rinsed off with PBS in the next morning. A goat-anti-rabbit IgG H&L (HRP) (1:1000, ab6721, Abcam, Cambridge shire, England) and goat-anti-mouse IgG H&L (HRP) (1:2000, ab205719, Abcam, Cambridge shire, England) antibodies were applied as the secondary antibodies and incubated in a humidified chamber for 1 h at room temperature. The excess secondary antibody was rinsed off with PBS, and a DAB color solution (DA1015, Solarbio, Beijing, China) was then prepared fresh for proper color development, after which the sections were washed with distilled water. The sections were placed into hematoxylin staining solution for 3 min and washed with distilled water for 15 min, followed by conventional dehydration and transparent and neutral resin sealing. The images were captured using a microscope equipped with a DP71 camera (BX51, Olympus, Tokyo, Japan).

### Statistical analysis

Data are expressed as the mean ± SEM from at least 3 independent experiments. The distance and number of cell migrations were analyzed using the image analysis software of Image-Pro Plus 6.0. For measurement data with equal variances, one-way ANOVA was performed to determine the differences between groups [29]. For data with unequal variances, the Wilcoxon rank-sum test was used. The data were processed with SPSS 22.0 software (SPSS, Inc., Chicago, IL, USA) and visualized using GraphPad Prism 6.0 (GraphPad Software, Inc., La Jolla, CA, USA). *P*-values < 0.05 were considered significant.

## Results

### Influences of rhEGF on the proliferation of fibroblasts and epidermal cells

**Fig. 1:**
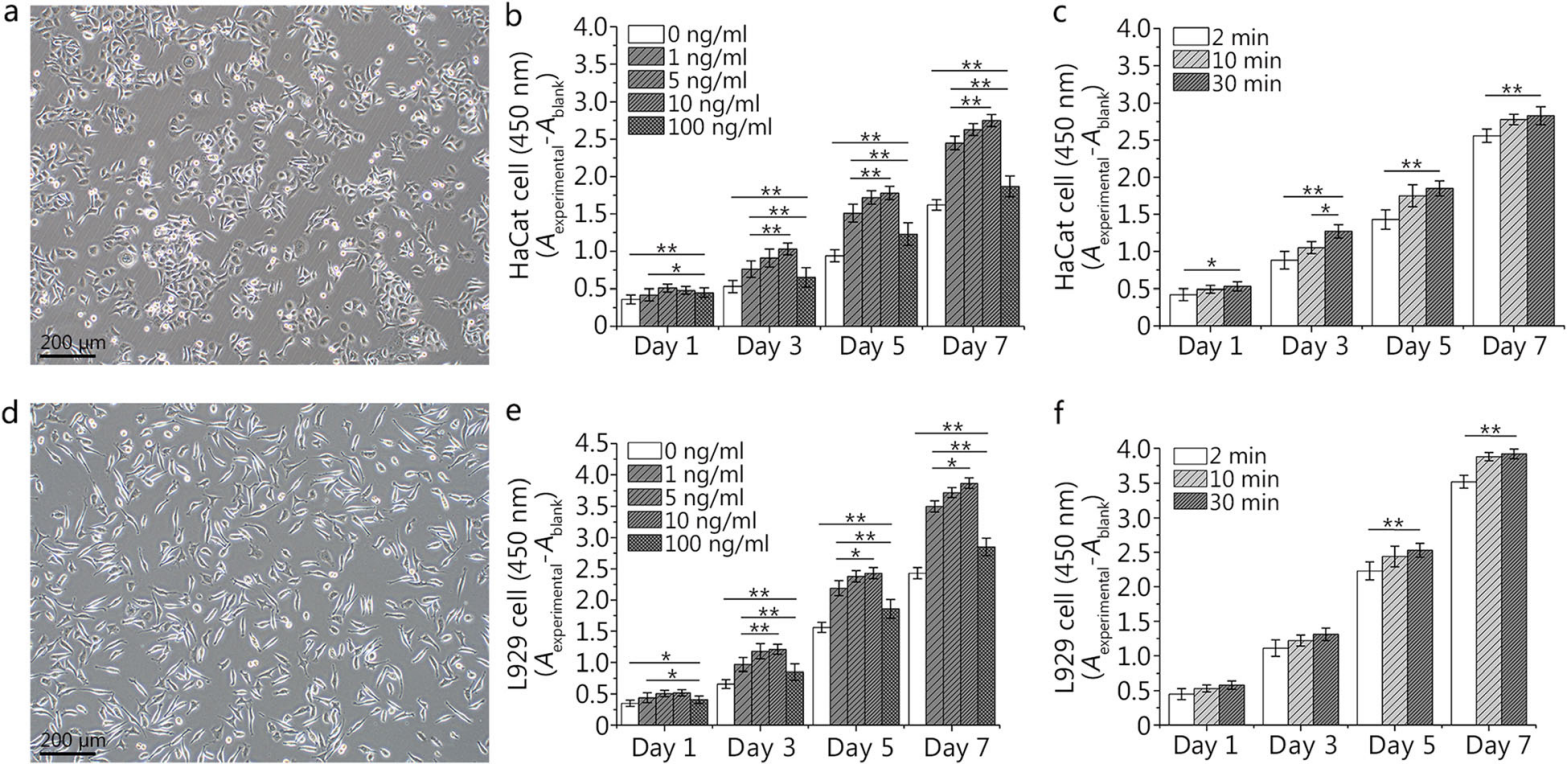
Effects of different concentrations and durations of epidermal growth factor (EGF) on the proliferation of HaCaT and L929 cells. **a**. Observation of HaCaT cells at day 2 under an inverted microscope. **b**. The effects of different concentrations (0, 1, 5, 10, and 100 ng/ml) of EGF on the proliferation of HaCaT cells at different time. **c**. The effects of different duration (2, 10, and 30 min) and a set concentration (10 ng/ml) of EGF on the proliferation of HaCaT cells at different time. **d**. Observation of L929 cells at day 2 under an inverted microscope. **e**. The effects of different concentrations (0, 1, 5, 10, and 100 ng/ml) of EGF on the proliferation of L929 cells at different time. **f**. The effects of different duration (2, 10, and 30 min) and a set concentration (10 ng/ml) of EGF on the proliferation of L929 cells at different time. Data are shown as means ± SD, *P < 0.05, **P < 0.01

We first cultivated two important cell lines during wound healing and adjusted their cell status to achieve the best conditions. As shown in Fig. 1a, HaCaT cells are approximately elliptical in shape and grow in small islands. Compared with HaCaT cells, fibroblasts, L929 cells, have a typical spindle type, and grow scattered (Fig. 1d). We evaluated the effect of EGF on cell proliferation by CCK-8 assay, and the absorbance indirectly reflects the change in cell number. With the prolongation of the culture time, the number of cells in each EGF concentration group (0, 1, 5, 10, and 100 ng/ml) gradually increased (Fig. 1b). On the first day of culture, although the absorbances of the four groups containing EGF were higher than that of the blank group, there was no statistical significance between the three experimental groups (5, 10, and 100 ng/ml). On the 3rd, 5th and 7th days of culture, the cells in each group showed the same trend. The experimental group of 10 ng/ml had the highest absorbance, but there was no statistical significance between the two experimental groups (5 and 10 ng/ml). Compared with HaCaT cells, L929 cells showed the same trend and generally had a higher absorbance (Fig. 1e).

Based on an EGF concentration of 10 ng/ml, we adjusted the incubation time (Fig. 1c). The two experimental groups (10 and 30 min) had higher absorbance than that of the 2-min group. Although there was statistical significance between the two experimental groups (10 and 30 min) on the 3rd day of culture, there was no statistical significance at the other times of culture. Compared with HaCaT cells, all the experimental groups (2, 10, and 30 min) of L929 cells had higher absorbance values. The two experimental groups (10 and 30 min) of L929 cells had no statistical significance at any culture times (Fig. 1f). Additionally, there was no statistical significance among all groups on the 1st and 3rd days of culture.

### Influences of rhEGF on the migration of fibroblasts and epidermal cells

**Fig. 2:**
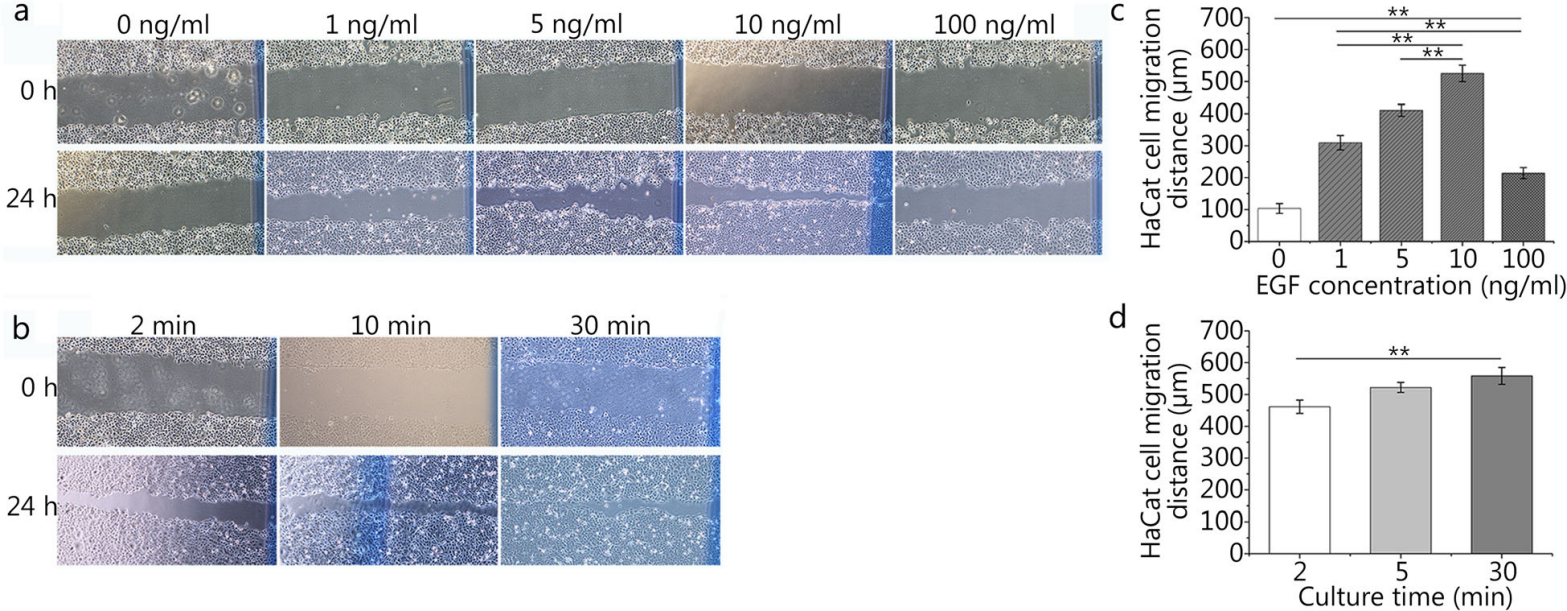
Effects of different concentrations and durations of epidermal growth factor (EGF) on HaCaT cell migration. **a** and **c**. The effects of different concentrations (0, 1, 5, 10, and 100 ng/ml) of EGF on the migration distance of HaCaT cells (0 ng/ml: 103.37 ± 15.37 μm, 1 ng/ml: 309.34 ± 22.68 μm, 5 ng/ml: 410.63 ± 18.60 μm, 10 ng/ml: 525.24 ± 25.79 μm, 100 ng/ml: 213.94 ± 17.40 μm). **b** and **d.** The effects of different durations (2, 10, and 30 min) and a set concentration (10 ng/ml) of EGF on the migration distance of HaCaT cells (2 min: 461.46 ± 20.84 μm, 10 min: 522.18 ± 15.59 μm, 30 min: 558.35 ± 26.68 μm). The scale bar is 500 μm. Data are shown as means ± SD, **P < 0.01

**Fig. 3:**
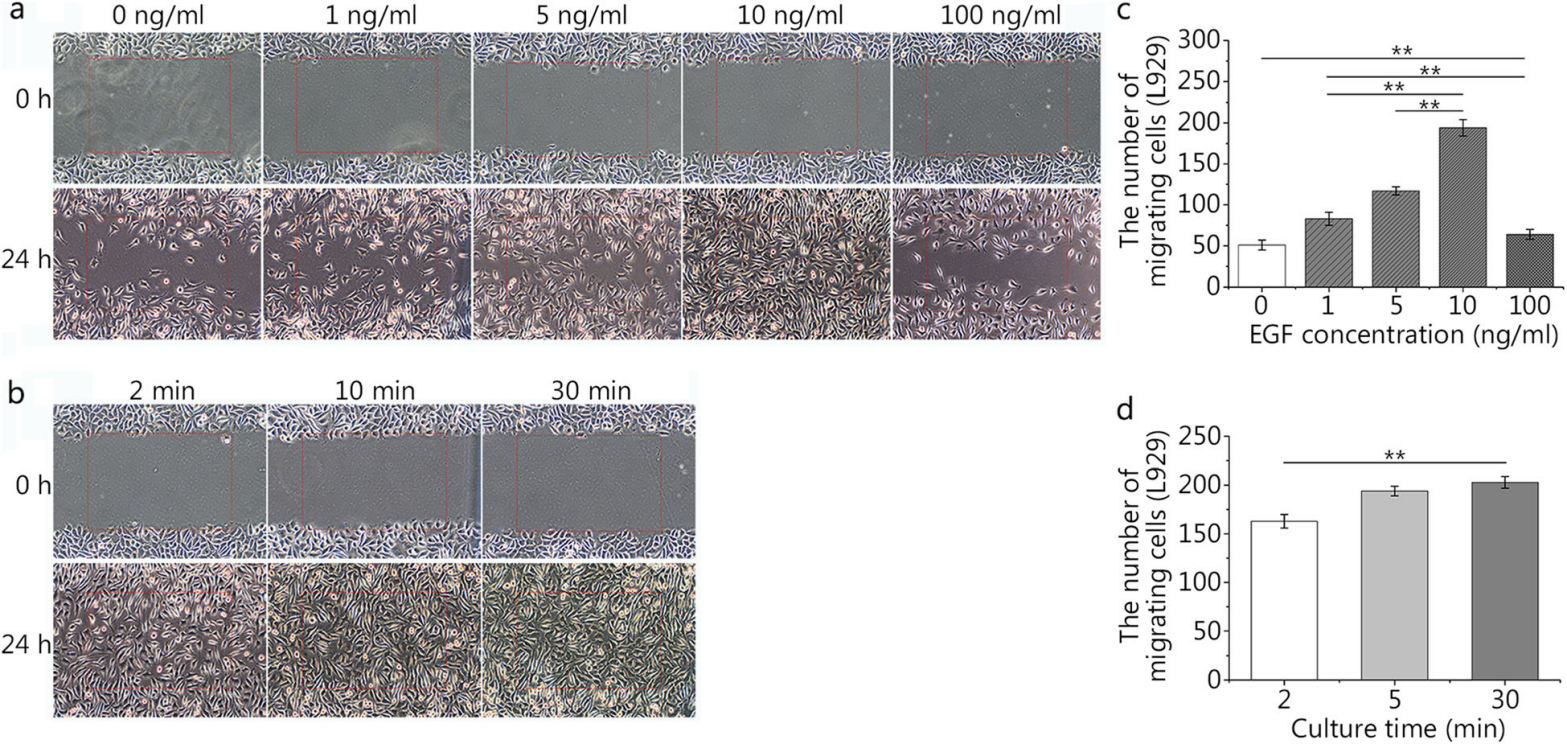
Effects of different concentrations and durations of epidermal growth factor (EGF) on L929 cell migration. **a** and **c**. The effects of different concentrations (0, 1, 5, 10, and 100 ng/ml) of EGF on the migration number of L929 cells (0 ng/ml: 51 ± 6, 1 ng/ml: 83 ± 8, 5 ng/ml: 117 ± 5, 10 ng/ml: 194 ± 10, 100 ng/ml: 64 ± 6). **b** and **d**. The effects of different duration (2, 10, and 30 min) and a set concentration (10 ng/ml) of EGF on the migration number of L929 cells (2 min: 163 ± 7, 10 min: 194 ± 5, 30 min: 203 ± 6). The scale bar is 500 μm. Data are shown as means ± SD, **P < 0.01

The migration of HaCaT cells and fibroblasts from the surrounding epidermis may play an important role in wound closure. The scratch wound assay revealed that EGF at a concentration of 10 ng/ml can promote the migration of HaCaT cells to the greatest extent (Fig. 2a), and the 10 ng/ml group had the greatest cell migration distance at 24 h of culture (Fig. 2c). There was statistical significance between the experimental groups (1, 5, 10, and 100 ng/ml) and the blank group (0 ng/ml). According to the results of the pre-experiment, we know that fibroblasts have a faster migration speed (data not shown). As a result, we chose the number of migrated cells in the scratch assay as the evaluation index rather than the distance. Compared with HaCaT cells, L929 cells showed the same trend, and the 10 ng/ml group had the greatest number of migrating cells at 24 h of culture (Fig. 3a and c).

Based on an EGF concentration of 10 ng/ml, we chose the different incubation times (2, 10, and 30 min) in the scratch wound assay (Fig. 2b). The two experimental groups (10 and 30 min) had the farthest distance of cell migration at 24 h of culture compared with the 2-min group (Fig. 2d). There was no statistical significance between the two experimental groups (10 and 30 min). Compared with HaCaT cells, L929 cells showed the same trend (Fig. 3b and d). The 30-min group had the greatest number of migrating cells at 24 h of culture, and there was no statistical significance between the two experimental groups (10 and 30 min).

### Diffusion effects of different administration methods

**Fig. 4:**
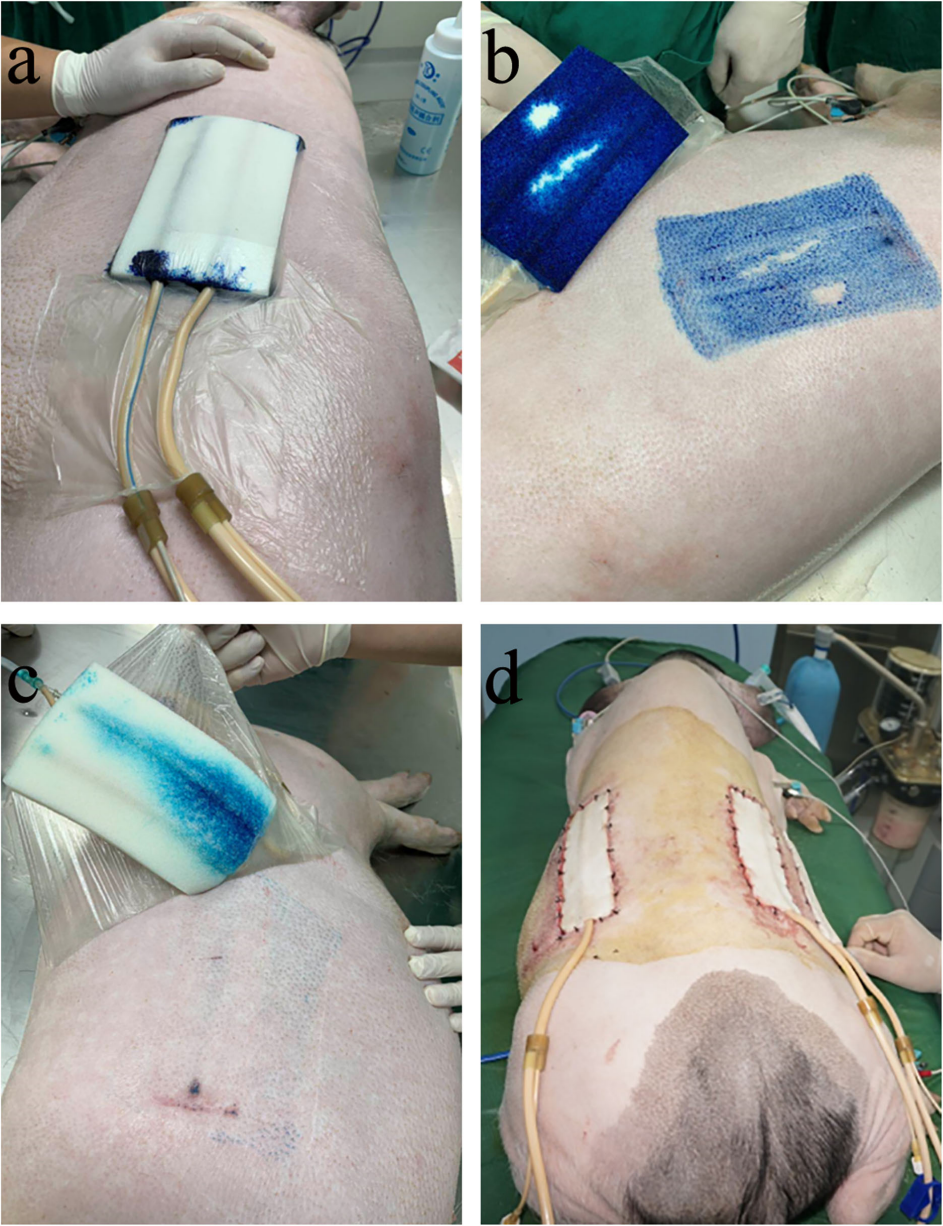
Effects of different administration methods on drug dispersion during vacuum sealing drainage (VSD) treatment and a trauma model in Bama pigs. **a**. Image of the opening the VSD negative pressure device for suction after injection with a 15-ml syringe. **b**. Image of the dispersion effect after injection with the 15-ml syringe. **c.** Image of the dispersion effect after injection with an infusion set. **d.** The trauma model of the Bama pig after surgical operation using VSD and epidermal growth factor (EGF)

To evaluate the diffusion effects of different administration methods in wound treatment with VSD, we injected Methylene Blue Solution using two infusion sets and a 15-ml syringe. Figure 4a showed the opening of the VSD negative pressure device for suction on the pig’s surgical area after injection with the 15-ml syringe. Figure 4b shows the diffusion effect of injection using the 15-ml syringe. Figure 4c shows the diffusion effect of the instillation method using the infusion set. As shown in Fig. 4, compared with the infusion set method, the 15-ml syringe method had a better diffusion effect. Figure 4d shows the postoperative photo of VSD combined with EGF in the treatment of a full-thickness skin defect of a Bama pig.

### Effect of EGF combined with VSD on wound healing

**Fig. 5:**
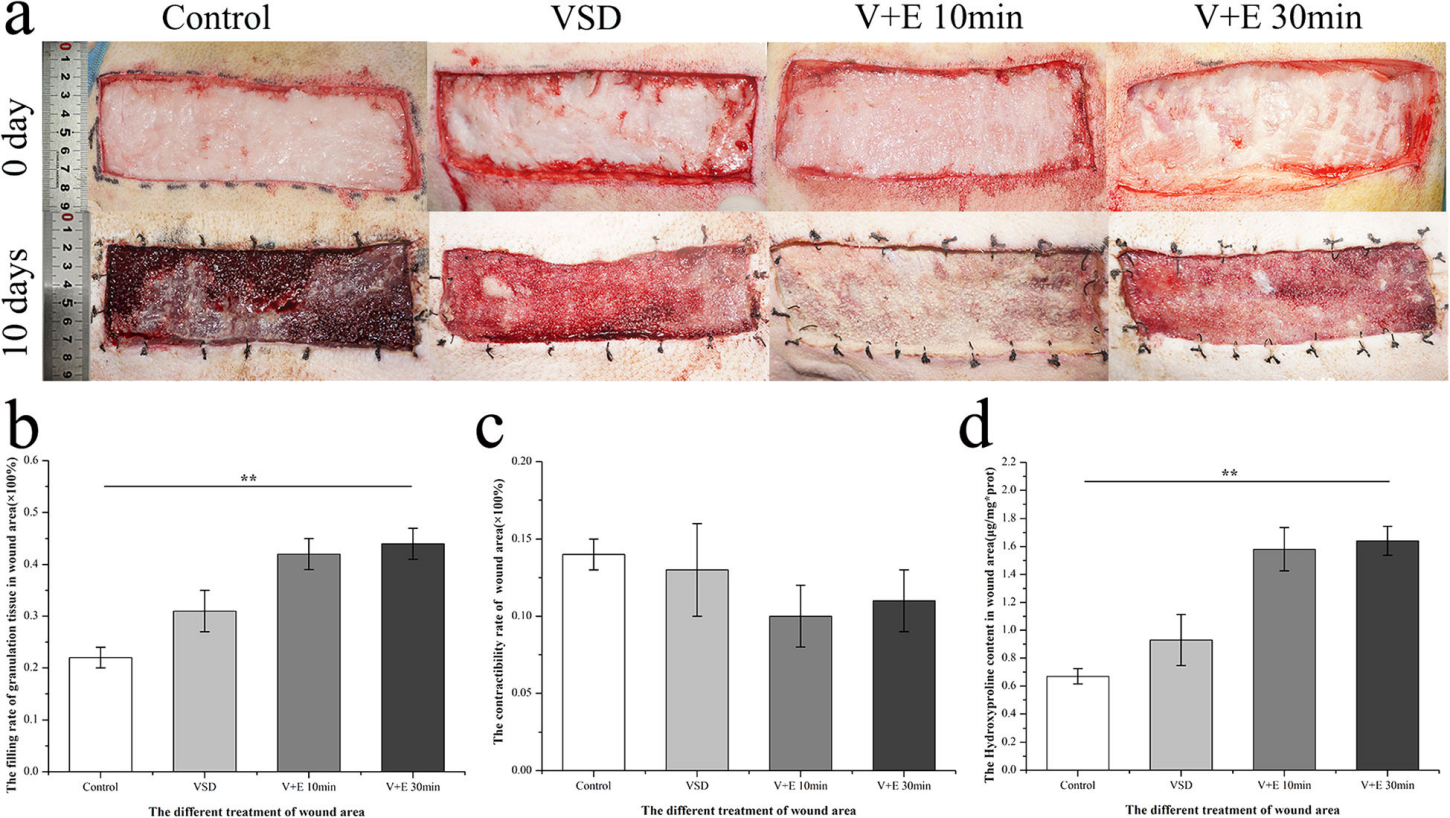
General view of the postoperative wound and evaluation of the effect of epidermal growth factor (EGF) combined with vacuum sealing drainage (VSD) in the treatment of wounds. **a**. General views of the postoperative wound and the wound 10 d after the operation. **b**. The filling rate of granulation tissue in the wound area 10 d after the operation (Control: 0.22 ± 0.02, VSD: 0.31 ± 0.04, V + E 10 min: 0.42 ± 0.03, V + E 30 min: 0.44 ± 0.03). **c**. The contractibility rate of the wound area 10 d after the operation (Control: 0.14 ± 0.01, VSD: 0.13 ± 0.03, V + E 10 min: 0.10 ± 0.02, V + E 30 min: 0.11 ± 0.02). **d**. The hydroxyproline content in the wound area 10 d after the operation (Control: 0.67 ± 0.05 μg/mg*prot, VSD: 0.93 ± 0.18 μg/mg*prot, V + E 10 min: 1.58 ± 0.16 μg/mg*prot, V + E 30 min: 1.64 ± 0.10 μg/mg*prot). Data are shown as means ± SD, **P < 0.01

To evaluate the effect of EGF combined with VSD on wound healing, we calculated the filling rate of granulation tissue of the wound area, the contractibility rate of the wound area and the hydroxyproline content of the wound area for each experimental group 10 d after operation. The upper half of Fig. 5a shows a general view of the postoperative full-thickness skin defect modeling, and the lower half of Fig. 5a shows a general view of 10 d after operation. By comparison, we can see that the wounds in each group have different degrees of healing. Therefore, we analyzed the relevant indicators using Image-Pro Plus 6.0 software. With regard to the filling rate of granulation tissue, the experimental groups (VSD, V + E 10 min, V + E 30 min) had greater values than the Control group (Fig. 5b). Meanwhile, there was no statistical significance between the two experimental groups (V + E 10 min, V + E 30 min). Compared with the indicator of the filling rate of granulation tissue, the indicator of hydroxyproline content in the wound area showed the same trend (Fig. 5d). However, there was no statistical significance between the groups for the indicators of the contractibility rate of the wound area, and two experimental groups (V + E 10 min and V + E 30 min) had lower values (Fig. 5c).

### Histological analysis of wound healing

**Fig. 6:**
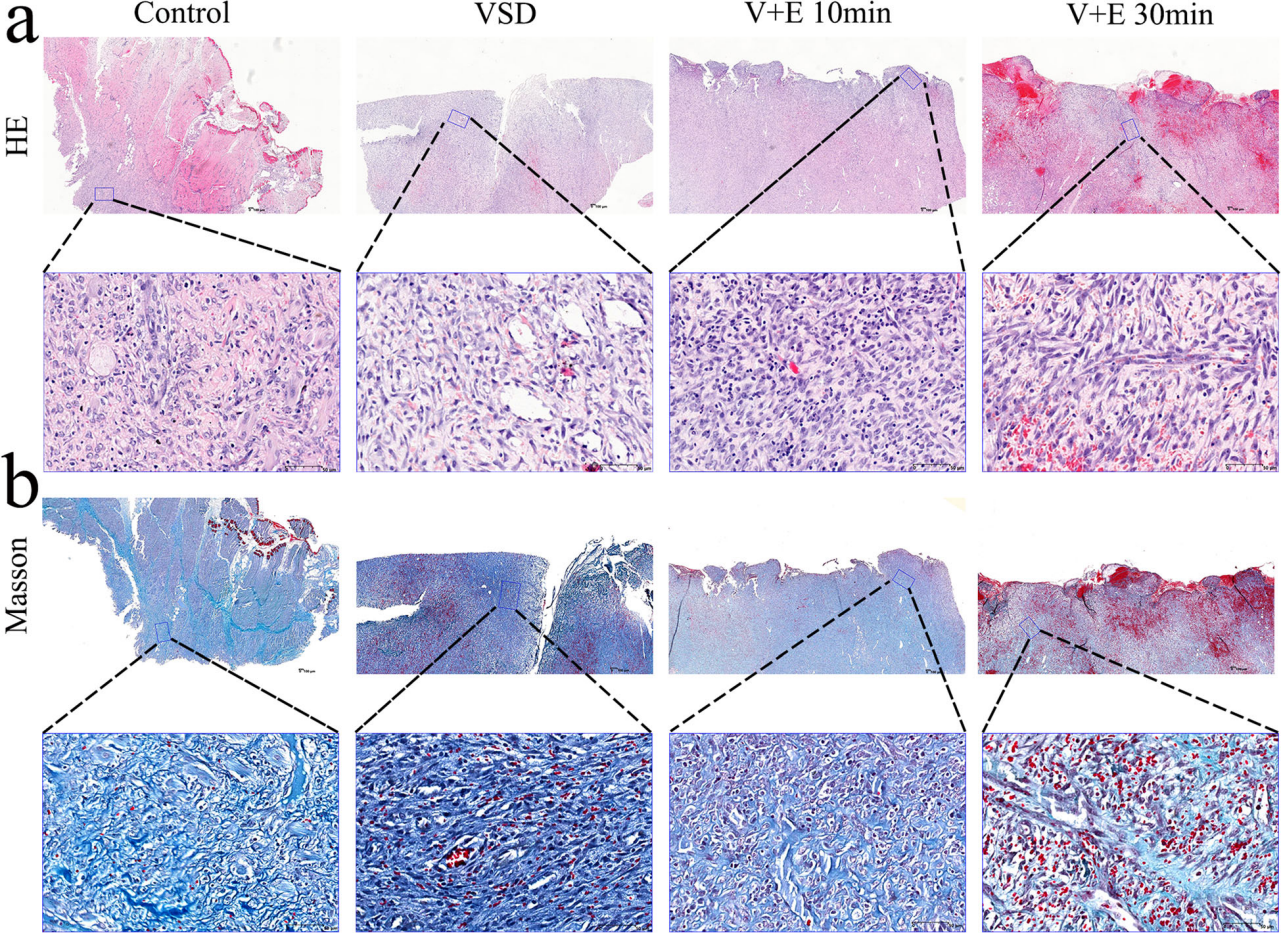
Histological evaluation of epidermal growth factor (EGF) combined with vacuum sealing drainage (VSD) in the treatment of wounds. **a.** HE staining of the wound 10 d after operation; the following picture is an enlargement of the local area from the above picture. **b**. Masson staining of the wound 10 d after the operation; the following picture is the enlargement of the local area from the above picture. The scale bar in the global image above is 500 μm, and the scale bar in the enlarged image below is 70 μm

The results of HE and Masson staining of wound tissue in each group (Fig. 6) showed that, with regard to the Control group, the injury involved the muscularis, there was no epidermal healing, fibroblast proliferation was active (++), and the arrangement of new collagen fibers was disordered. Neovascularization (+) accompanied by inflammatory cell infiltration (++) can be seen. With regard to the VSD group, the injury involved subcutaneous tissue, there was no epidermal healing, fibroblast proliferation was active (+++), and a large number of new collagen fibers were in disorder. A large amount of neovascularization (++) with inflammatory cell (+) infiltration can be seen. With regard to the V + E 10 min group, the injury involved the dermal layer but there was still dermal residue, and no new epidermis was found in the wound. The proliferation of new fibroblasts was active (+++), and a large number of new collagen fibers were in disorder. A large amount of neovascularization (++) was accompanied by a large amount of inflammatory cell infiltration (++). With regard to the V + E 30 min group, the injury involved subcutaneous tissue, necrosis was seen at the wound, and no epidermal healing was observed. Scar formation occurred, hemorrhage was visible, fibroblast proliferation was active (+++), and a large number of new collagen fibers were arranged in disorder. Large amounts of neovascularization (++) and inflammatory cell infiltration (++) were observed.

### Immunohistochemical assessment of collagen deposition

**Fig. 7:**
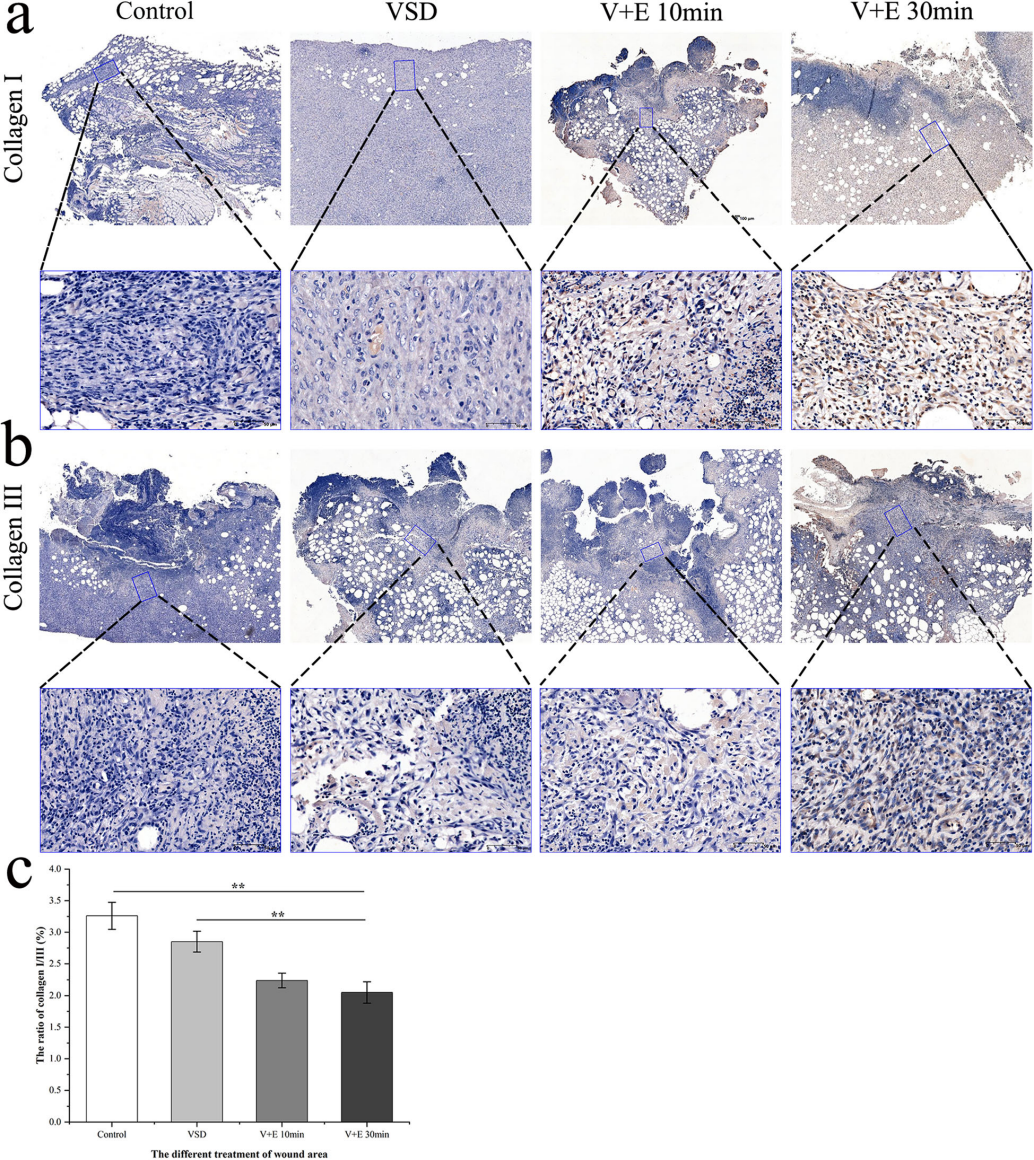
Immunohistochemical evaluation of epidermal growth factor (EGF) combined with vacuum sealing drainage (VSD) in the treatment of wounds. **a**. Immunohistochemical staining of collagen I in the wound 10 d after the operation; the following picture is an enlargement of the local area from the above picture. **b.** Immunohistochemical staining of collagen III in the wound 10 d after the operation; the following picture is an enlargement of the local area from the above picture. **c.** The ratio of collagen I/III in the wound area 10 d after the operation (Control: 3.26 ± 0.22, VSD: 2.85 ± 0.17, V + E 10 min: 2.24 ± 0.12, V + E 30 min: 2.05 ± 0.17). The scale bar in the global image above is 500 μm, and the scale bar in the enlarged image below is 70 μm. Data are shown as means ± SD, **P < 0.01

The results of immunohistochemical of collagen I and collagen III staining in each group showed that in the Control and VSD groups, there was more type I collagen in the wound tissue (Fig. 7a), while the content of type III collagen was relatively low (Fig. 7b). However, in the V + E 10 min and V + E 30 min groups, there was more type III collagen in the wound tissue, while the content of type I collagen was relatively low. The contents of collagen I and collagen III in the skin are high. Collagen III is newly synthesized collagen, which plays an important role in the process of wound repair. Through analysis using Image-Pro Plus 6.0 software, we drew the following conclusions (Fig. 7c): Compared with the Control group, the V + E 10 min and V + E 30 min groups had lower ratios of collagen I/III in the wound area, but there was no statistical significance between the two experimental groups.

## Discussion

More than half a century ago, Dr. Cohen discovered EGF [30]. After a series of studies, EGF was isolated, purified and identified. By binding and activating EGFR, EGF can induce many biological reactions, including cell proliferation, differentiation and migration [31], and its signal transduction plays a regulatory role in normal development, as well as pathophysiological events, such as tissue repair, including ulcer/wound healing [32, 33]. Wound healing is a complex process, including mechanical coupling induced motion between cells and rhEGF mediated biochemical stimulation of migration and motility in close surrounding [34, 35]. Specifically, after binding to EGF, EGFR enters cells mainly via clathrin-mediated endocytosis (CME) [36]. Here, the receptor has two fates: one in which is circulated to the cell surface to continue to play a role; the other in which is further transported to the late endosomes and lysosomes for degradation [37]. After a series of studies, Sigismund et al [26] found that EGFR internalized through CME is not intended for degradation, but rather circulates to the cell surface. In contrast, clathrin-independent internalization (non-clathrin endocytosis, NCE) preferentially degrades the receptor. Meanwhile, compared with a low EGF concentration (1.5 ng/ml, when CME is predominant and ~ 30% of the internalized ligand is degraded), at a high EGF concentration (100 ng/ml), ~ 55% of the ligand was degraded (~ 60% and ~ 40% EGF enters through the CME and NCE, respectively). This observation also explains why the concentrations of EGF in clinical application is not as high as possible.

In our study (Figs. 1, 2 and 3), we also found that of all the EGF concentration groups (0, 1, 5, 10, and 100 ng/ml), the 10 ng/ml group could promote the proliferation and migration of HaCaT and L929 cells to the greatest extent. It may be that the receptor is partially degraded under the condition of a high EGF concentration, and the results of the high concentration group (100 ng/ml) were only better than those of the blank group (0 ng/ml) and were far less than those of the other experimental groups (1, 5, and 10 ng/ml). Meanwhile, Sigismund et al [26] also found that the content of EGFR on the cell surface decreased by 50% after 6 min of EGF treatment; the content of EGFR decreased by 80% 30 min later. Interestingly, in this study (Figs. 1, 2 and 3), we found that there was no statistical significance between the two groups (10 and 30 min) in promoting the proliferation and migration of HaCaT and L929 cells. EGFR on the cell surface may decrease to very low levels after 10 min. As time goes on, fewer signals are transmitted into the cell through EGFR, which will not produce statistical significance.

Timely and effective treatment of skin wounds is essential to prevent microbial infection and skin water loss, and to accelerate wound repair. In medical institutions, contaminated wounds are debrided well. Meanwhile, to maintain the best condition of the wound, proper dressing or covering is usually required, which is expected to cover the entire wound area to protect damaged tissue and promote healing. Relevant studies have shown that a wet state can promote wound healing compared with a dry state [38, 39]. VSD is carried out in a closed system; the negative pressure drainage system can not only maintain the wet state, but can also quickly remove any exudate and necrotic tissue. Several recent related studies [40] have shown that VSD treatment can effectively shorten the wound healing time, reduce the pain caused by frequent drug changes, and effectively avoid cross-infection. Continuous negative pressure promotes the flow of body fluid and exudate from the wound to the drainage tube, which provides effective and continuous auxiliary power for blood circulation, thus promoting the growth of granulation tissue in the wound [41]. In our study (Fig. 5), compared with the Control group (routine dressing change), the VSD group had a higher filling rate of granulation tissue in the wound area. Interestingly, although there was no statistical significance between the groups, compared with the Control and VSD groups, the two experimental groups (V + E 10 min and V + E 30 min) had lower contractibility rates in the wound area. We speculate that EGF can promote the growth of granulation tissue while reducing the contraction of the wound, which may reduce scar formation.

For different diseases and wounds with different courses, the mechanism and usage of EGF may be different, especially for acute and chronic wounds. Exogenous EGF is easily degraded in a chronic wound environment, which limits its application in the process of chronic wound healing [24]. Kim et al [42] found that a hyaluronate-EGF conjugate patch plays an important role in chronic wound healing. Meanwhile, the application of new nano-biomaterials has greatly promoted the development in the field of wound healing [43]. Recently, Garcia-Orue et al [44] found that a PLGA nanofibrous membrane that contains rhEGF improved fibroblast proliferation and significantly accelerated wound closure and reepithelization in an in vivo full-thickness wound healing assay carried out in mice. However, with regard to acute wounds, there is no large amount of matrix metalloproteinases (MMPs) to degrade EGF and less exudate [45]. Therefore, we chose to apply rhEGF combined with VSD directly to repair the wounds made in this study.

Many studies have shown that the local concentration of EGF needs to be sufficiently high and maintained for a long enough time to effectively promote wound healing [46, 47]. Brown et al [48] applied a silver sulfadiazine cream containing EGF (10 μg/ml) to partial-thickness skin wounds of 12 patients who required skin grafting for either burns or reconstructive surgery. The concentration of rhEGF for external use recommended by the Chinese Pharmacopoeia in 2020 is 5 μg/ml. Due to the exudate secreted from the wound and the presence of different amounts of degrading enzymes, the concentration of rhEGF in vivo must be higher than that in cell experiments (10 ng/ml). Therefore, the concentration of rhEGF used in our experiment was 4 μg/ml (30 ml, 15 cm in length × 5 cm in width × 1.5 cm in depth). Considering that there was no statistical significance between the two experimental groups (V + E 10 min and V + E 30 min) and operator should avoid possible wound infections caused by prolonged operation, the recommended EGF action time based on our study is 10 min.

## Conclusions

A rhEGF concentration of 10 ng/ml that can promote the proliferation and migration of epithelial cells and fibroblasts to the greatest extent in vitro. VSD combined with EGF, kept in place for 10 min and then washed, can promote collagen deposition and wound healing better in vivo. This promising treatment strategy can be applied to acute skin wounds caused by burns or injury.

## Data Availability

The datasets used and/or analyzed in the current study are available from the corresponding author upon reasonable request.

## Abbreviations

VSD: Vacuum sealing drainage
EGF: Epidermal growth factor
rhEGF: Recombinant human epidermal growth factor
ECM: Extracellular matrix
EGFR: Epidermal growth factor receptor
RTKs: Receptor tyrosine kinases
FBS: Fetal bovine serum
CME: Clathrin-mediated endocytosis
NCE: Non-clathrin endocytosis
